# The Association Between the Use of Digital Health Management Tools and Subjective Well-Being Among Older Adults

**DOI:** 10.1093/geroni/igab046.1914

**Published:** 2021-12-17

**Authors:** Haowei Wang, Mai See Yang, Yong Kyung Choi

**Affiliations:** 1 The Pennsylvania State University, University Park, Pennsylvania, United States; 2 UC Davis, UC Davis, California, United States; 3 University of California Davis School of Medicine, Sacramento, California, United States

## Abstract

This study aims to examine the association between the use of digital health management tools and subjective well-being in later life. Research is limited about technology use (e.g., participation in online wellness program, finding medical information, using devices to monitor health) among community dwelling older adults. This study used data from the Health and Retirement study 2012 Module “Technology Use: Barriers and Benefits” (N = 1,416). We used multiple regression methods to test the association between technology use and subjective well-being (i.e., self-rated health, life satisfaction, and depressive symptoms). Over half of the participants reported using technology (58%). The mean age for this group was 68.7 (SD 9.6). Majority of the respondents were female (55%). About 18% were non-Hispanic Blacks, 2% were non-Hispanic other, 11% were Hispanic, and 68% were non-Hispanic Whites. For this sample of technology users, the usage of digital health management included online exercise programs (16%), online wellness programs or health monitoring programs (7%), searching for medical and health information online (43%), digital devices to monitor health (31%), and physical activity-based video game such as Wii Fit (7%). Over 88% of the sample have used at least one of these formats to monitor their health. Results from regression models suggested that the use of any digital health management tools was related to fewer depressive symptoms and better self-reported health. Findings from this study provide insight into how digital health management can protect older adults from poor subjective well-being in later life.

